# Apocynin Prevents Anxiety-Like Behavior and Histone Deacetylases Overexpression Induced by Sub-Chronic Stress in Mice

**DOI:** 10.3390/biom11060885

**Published:** 2021-06-15

**Authors:** Silvia S. Barbieri, Leonardo Sandrini, Laura Musazzi, Maurizio Popoli, Alessandro Ieraci

**Affiliations:** 1 Unit of Brain-Heart Axis: Cellular and Molecular Mechanisms, Centro Cardiologico Monzino IRCCS, 20138 Milan, Italy; silvia.barbieri@ccfm.it (S.S.B.); leonardo.sandrini@ccfm.it (L.S.); 2 Department of Medicine and Surgery, University of Milano-Bicocca, 20900 Monza, Italy; laura.musazzi@unimib.it; 3 Laboratory of Neuropsychopharmacology and Functional Neurogenomics, Dipartimento di Scienze Farmaceutiche, University of Milan, 20133 Milan, Italy; maurizio.popoli@unimi.it

**Keywords:** anxiety disorders, apocynin, oxidative stress, ROS, HDAC, epigenetic, NADPH oxidase

## Abstract

Anxiety disorders are common mental health diseases affecting up to 7% of people around the world. Stress is considered one of the major environmental risk factors to promote anxiety disorders through mechanisms involving epigenetic changes. Moreover, alteration in redox balance and increased reactive oxygen species (ROS) production have been detected in anxiety patients and in stressed-animal models of anxiety. Here we tested if the administration of apocynin, a natural origin antioxidant, may prevent the anxiety-like phenotype and reduction of histone acetylation induced by a subchronic forced swimming stress (FSS) paradigm. We found that apocynin prevented the enhanced latency time in the novelty-suppressed feeding test, and the production of malondialdehyde induced by FSS. Moreover, apocynin was able to block the upregulation of p47phox, a key subunit of the NADPH oxidase complex. Finally, apocynin prevented the rise of hippocampal *Hdac1*, *Hdac4* and *Hdac5*, and the reduction of histone-3 acetylation levels promoted by FSS exposure. In conclusion, our results provide evidence that apocynin reduces the deleterious effect of stress and suggests that oxidative stress may regulate epigenetic mechanisms.

## 1. Introduction

Anxiety, a neuropsychiatric disorder characterized by high arousal and elevated vigilance in the absence of instantaneous risks [1], is a severe and life-threatening psychiatric disease affecting millions of people, causing heavy social and economic burdens worldwide [2]. The etiopathogenesis of anxiety is still not fully understood, but it is supposed that both genetic variants and environmental factors contribute to the onset of these disorders. In particular, stressful life events are considered one of the main risk factors for neuropsychiatric diseases [3,4]

Molecular mechanisms underlying the stress-induced alterations in brain structure and function associated with psychiatric disorders are still a matter of discussion. Accumulating evidence suggests that stress-dependent aberrant transcriptional regulation in the brain plays a key role in the pathophysiology of anxiety [5,6,7,8]. Chromatin remodeling, which is mainly attributed to histone posttranslational acetylation and methylation, is recognized as an important mechanism involved in the regulation of gene expression [9,10]. Histone acetylation is generally associated with active gene expression and is regulated by the opposite activities of histone acetyltransferase and histone deacetylases (HDACs) [11]. Intriguingly, altered expression of HDACs and histone acetylation levels have been reported in the postmortem brain tissue of patients suffering from mood and anxiety disorders [12]. Moreover, increased *Hdacs* mRNA levels in leucocytes have been observed in depressed patients [13]. Similarly, in preclinical animal models of stress-related disorders, an increase of HDACs expression has been observed paralleled by histone acetylation reduction [6,12,14,15,16]. However, the mechanisms by which stress regulates HDACs expression remains to be elucidated.

Psychiatric conditions have also been consistently associated with an increase in oxidative stress [17,18,19,20]. Oxidative stress results from an imbalance between reactive oxygen species (ROS) production and the capability of cell scavenging systems to detoxify ROS. The brain is particularly vulnerable to oxidative damage caused by ROS, as it consumes large quantities of oxygen and has abundant lipid content, but relative scarcity of antioxidant compounds [19]. In particular, the hippocampus, amygdala and prefrontal cortex seem to be the brain areas mostly affected by ROS, leading to behavioral and cognitive alterations [21]. In fact, in postmortem tissues of depressed patients and of stress-based animal models, increased oxidative stress damage (lipid peroxidation, DNA damage and protein oxidation) has been observed in these brain areas [21,22,23]. Remarkably, it has been proposed that ROS may be involved in the regulation of gene expression as they can alter epigenetic mechanisms such as DNA methylation and histone modifications [24]. There are multiple potential sources of ROS in mammalian cells and, among them, nicotinamide dinucleotide adenine dinucleotide phosphate (NADPH) oxidase plays an important role, as it is dedicated to the specific production of superoxide. NADPH oxidase is a membrane-bound enzyme complex formed of three cytosolic subunits (p47phox, p67phox and p40phox), two membrane subunits (gp91phox and p22phox9) and Rho GTPase. Interestingly, chronic restraint stress increased hippocampal expression of p47phox and p67phox and promoted anxious and depressive-like phenotypes in mice, while in the p47phox heterozygous mice these behavioral alterations were attenuated [25,26].

Antioxidant compounds have been used to protect neurons against different type of damage [27,28,29,30]. Among them, apocynin (4-hidroxy-3-methoxyacetophenone) is one of the most frequently used as an NADPH oxidase inhibitor. The mechanism through which apocynin inhibits NADPH oxidase has not been fully elucidated, but it has been shown to involve the impairment of the translocation to the membrane of the cytosolic component p47phox of the NADPH-oxidase complex. Apocynin is a prodrug that, in the presence of H_2_O_2_ and myeloperoxidase, is converted to diapocynin, the actual active compound that inhibits NADPH oxidase by disrupting the interaction of p47phox with p22phox [31,32,33]. Although a large body of literature supports apocynin as a nonspecific NADPH oxidase inhibitor, there are some pieces of evidence also showing an intrinsic antioxidant activity [34]. For example, it has been reported that apocynin counteracts the reduction of superoxide dismutase (SOD), catalase (CAT) and glutathione (GSH) in an in vivo model of myocardial injury [35] and diabetes-associated cognitive decline [36]. However, the exact mechanism(s) of action of apocynin on antioxidant enzyme levels is not totally understood. Apocynin is an active principle with anti-inflammatory properties isolated from the root of the medical plant *Picrorhiza kurroa*, which can pass the blood-brain barrier [37]. 

We have previously reported that apocynin prevents the detrimental effect of subchronic forced-swimming stress (FSS) on thrombosis [38]. Here we aimed at studying if apocynin is also able to counteract the anxious-like behavioral phenotype induced by FSS, and to pinpoint the underlying mechanism(s).

## 2. Materials and Methods

### 2.1. Animals

Three-months old FVB male mice were purchased from Charles River and housed in a temperature-controlled room, 12 h light/dark cycle environment with ad libitum access to water and a standard chow diet. Mice were randomized in different groups. All animal handling and experimental procedures were performed in accordance with the European Community Council Directive (2010/63/EU) and were approved by Italian legislation on animal experimentation (Decreto Legislativo 116/1992). All efforts were made to minimize animal suffering and to diminish the numbers of mice used in this study. 

In a first set of experiments, mice were sacrificed the day after the last stress exposure and tissues were used for real-time PCR and Western blot analysis. In a second set of experiments, mice were tested in the Novelty-Suppressed Feeding test around 16 h after the last stress exposure and then sacrificed 24 h later. Collected tissues were used for lipid peroxidation and corticosterone quantification.

### 2.2. Drug Treatment

Mice were treated as previously described [38]. Briefly, apocynin (Sigma-Aldrich, Milan, Italy) was dissolved in dimethylsulfoxide (DMSO Sigma-Aldrich, Milan, Italy) (240 mg/mL) and then diluted 1:100 in saline solution or tap water. DMSO diluted 1:100 was used as vehicle. Apocynin (15 mg/kg) or vehicle were intraperitoneally (i.p.) injected 1 h before the first stress session and then added to the drinking water (2.4 mg/mL) for the following days. This amount corresponds to above 300 mg/kg, since our mice approximately drank 3–3.5 mL water/day.

### 2.3. Forced Swim Stress

The Forced Swim Stress (FSS) was conducted as previously described [38,39]. Briefly, single mice were placed in a glass beaker (height, 24 cm; diameter, 12 cm) containing 1500 mL of water (25 ± 2 °C) for 5 min twice a day for four consecutive days. After FSS, mice were immediately dried with a towel and returned to their home cage. During the first and the last day of the FSS, mice were videotaped and the total amount of immobility and swimming time were scored using the ANY-MAZE software (Stoelting, Purchased by Ugo Basile, Varese, Italy). 

### 2.4. Novelty-Suppressed Feeding Test

The Novelty-Suppressed Feeding (NSF) test was performed as previously described [40]. Briefly, in the center of a large rectangular arena (60 × 40 × 15 cm), filled with the wooden bedding, a regular food pellet was placed on a white paper platform. Before of the test, mice were food deprived for 24 h. An individual mouse was positioned in a corner and the latency to eat the pellet was scored. Immediately after the first bite, mice were transferred to their home cage and the quantity of food eaten during 5 min was weighed (home cage food consumption). 

### 2.5. Lipid Peroxidation Measurement

Lipid peroxidation was analyzed by measuring malondialdehyde (MDA) levels using thiobarbituric acid reactive substances (TBARS), a biochemical produced during the lipid peroxidation process [41]. Briefly, hippocampi and prefrontal cortex were homogenized in 1.15% KCl pH 7.4. Homogenized tissue (100 µL) or 50 µL of plasma were added to 150 µL of thiobarbituric acid (TBA) (8%), 150 µL of acetic acid (20%, pH 3.4) and 50 µL of Sodium Dodecyl Sulfate (SDS) (8.1%) and boiled for 60 min. Samples were chilled on ice and centrifuged at 13,200 rpm for 10 min. The absorbance of the collected supernatant was then measured at 530 nm using a microplate reader (Biorad, Milan, Italy).

### 2.6. RNA Isolation and Reverse Transcription Quantitative Real-Time PCR

Total RNA from the hippocampi was extracted using the Direct-zol™ RNA MiniPrep (Zymo Research, purchased by Euroclone, Milan, Italy) according to manufacturer’s instructions. cDNA was synthesized using the iScript kit (Biorad, Milan, Italy) according to manufacturer’s instructions. Quantitative Real-Time PCR analysis was performed on a CFX Connect Real Time System (Biorad) using the iTaq Universal SYBR Green supermix (Biorad), as previously described [42]. Primer sequences are described in Table 1. Relative expression of mRNA for the target genes was performed by the comparative C_T_ (ΔΔC_T_) method and normalized on *Rps8* and *Gapdh* housekeeping genes. Relative mRNA levels were expressed as fold-change. Primer’s specificity was established using the melting curve analysis.

### 2.7. Western Blot

Western blot analysis was performed as previously described [43]. Briefly, isolated hippocampus tissue was homogenized by using ice-cold RIPA buffer (15m mM NaCl, 5 mM Tris HCl, pH 7.4, 5 mM Ethylenediaminetetraacetic Acid (EDTA), 1% Triton X-100, 1% sodium deoxycholate, and 0.1% SDS) with Protease Inhibitor Cocktail (Sigma-Aldrich, Milan, Italy). Equal amounts of proteins (5 μg) were separated on SDS-PAGE gels and blotted to PVDF membranes (GE Healthcare, purchased by Euroclone). After being saturated in 5% milk in Tris Buffer Saline-Tween 20 (TBS-T), membranes were incubated with anti-acetyl H3 (1:2000 Millipore, purchased by Euroclone), anti-acetyl H4 (1:2000 Millipore, purchased by Euroclone) or anti-β-actin (1:20,000, mouse monoclonal, Sigma-Aldrich, Milan, Italy) antibodies. Membranes were extensively washed with TBS-T and then incubated with peroxidase-conjugated secondary antirabbit (1:3000, Sigma-Aldrich, Milan Italy) or with the fluorescent IRDye secondary anti-mouse antibody (LI-COR, purchased from Carlo Erba Reagents, Milan, Italy). Peroxidase immunoreactivity bands were revealed by chemiluminescence using ECL detection system (Biorad, Milan, Itay). Chemiluminescence and fluorescence membrane signals were scanned and quantified in an Odyssey LI-COR scanner (LI-COR, purchased by Carlo Erba Reagents Milan, Italy).

### 2.8. Statistical Analysis

Statistical analyses were performed using GraphPad Prism 6 (GraphPad Software, La Jolla, CA, USA). Data are shown as means ± standard error of the mean (SEM). Statistical analyses were made using a one-way analysis of variance (ANOVA) or a two-way ANOVA, and Turkey’s post hoc test was used for multiple comparison analysis when appropriate. Pearson’s r correlation was used to assess correlation between MDA levels and the anxiety-like phenotype.

## 3. Results

### 3.1. Apocynin Treatment Prevented the Enhancement of Anxiety-Like Phenotype Induced by FSS

Immobility time and total swimming distance were measured in the first day (stress session 1 and 2) and in the last day (stress session 7 and 8) of FSS. Immobility time increased (Time F_(3,92)_ = 39.31; *p* < 0.0001), while the swimming distance decreased over time (Distance F_(3,92)_ = 25.21; *p* < 0.0001). Moreover, in mice treated with apocynin, both the overall immobility time and swimming distance were reduced compared to vehicle-treated animals (Immobility time F_(3,92)_ = 9.02; *p* = 0.003; Total distance F_(3,92)_ = 7.211; *p* = 0.009). There was not a significant interaction effect between apocynin treatment and the immobility time or the total swimming distance.

**Figure 1:**
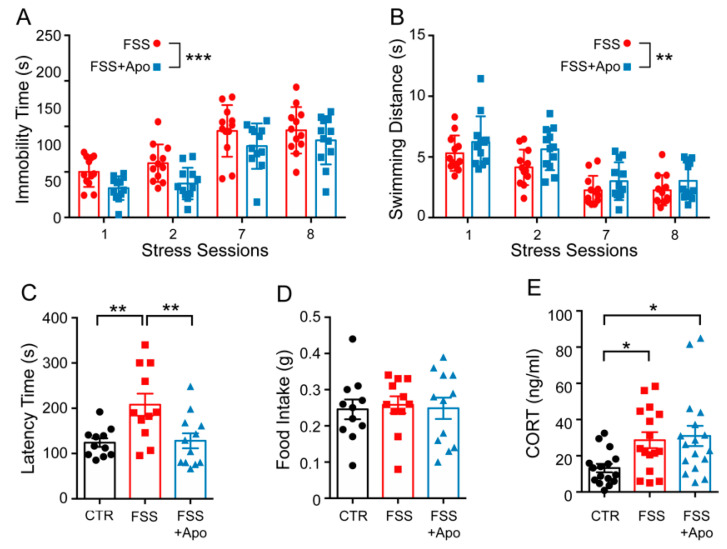
Apocynin prevents FSS-induced behavioral impairments. (**A**,**B**) Immobility time (**A**) and swimming distance (**B**) measured during the first and the last day of Forced Swimming Stress (FSS) exposure. Repeated two-way ANOVA. Data are presented as Mean ± SEM (*n* = 12 mice/group). (**C**,**D**) Novelty-Suppressed Feeding (NSF) test. Latency to bite the pellet food in a new large arena (**C**) and the amount of food consumed in the home cage (**D**). One-way ANOVA followed by Tukey’s post hoc analysis (*n* = 11–12 mice/group). (**E**) Plasmatic corticosterone levels measured 48 h after the last session of stress. One-way ANOVA followed by Tukey’s post hoc analysis. Data are presented as Mean ± SEM (*n* = 16–17 mice/group). * *p* < 0.05; ** *p* < 0.01; *** *p* < 0.001.

Mice subjected to FSS showed higher latency time in the Novelty-Suppressed Feeding test compared to control unstressed mice (*p* < 0.01), while apocynin administration during FSS exposure prevented the increased of latency time induced by FSS (*p* < 0.01) (Figure 1C). No significant difference was revealed among the groups for the total amount of pellets consumed in the home cage immediately after Novelty-Suppressed Feeding test (Figure 1D). Beneficial effects of apocynin were not dependent on the normalization of the hypothalamic-pituitary-adrenal axis response, because the stress-induced corticosterone increase was not modified by apocynin administration (Figure 1E)

### 3.2. Apocynin Treatment Prevented the Enhancement of Oxidative Stress Induced by FSS

**Figure 2:**
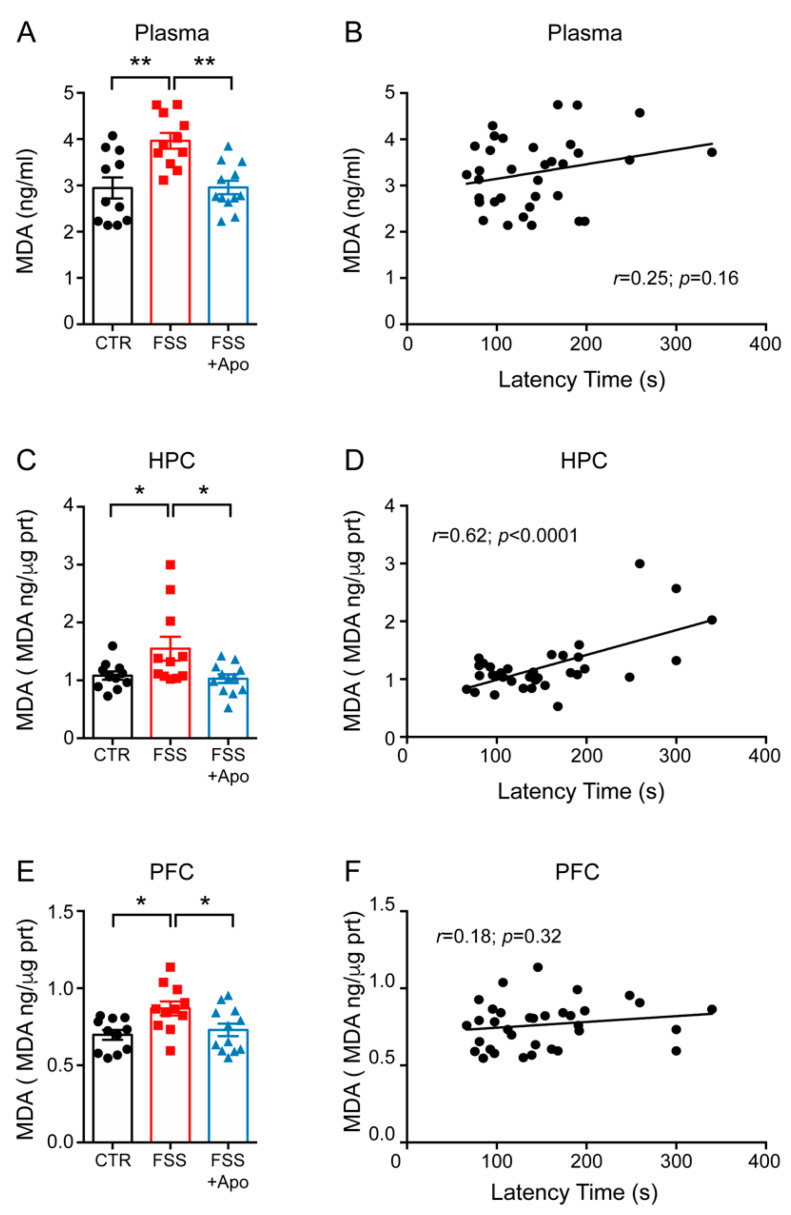
Apocynin prevents the FSS-induced increase of lipid peroxidation. (**A**,**C**,**E**) MDA levels measured in the plasma (**A**), hippocamps (HPC) (**C**) and prefrontal cortex (PFC) (**E**). One-way ANOVA followed by Tukey’s post hoc analysis. Data are presented as mean ± SEM (*n* = 11–12 mice/group). (**B**,**D**,**F**) Linear correlation between the latency to feed and the MDA levels in the plasma (**B**), HPC (**D**) and PFC (**F**). Pearson’s correlations (*n* = 11–12 mice/group). * *p* < 0.05; ** *p* < 0.01.

To investigate the effect of FSS on the levels of oxidative stress, we assessed the levels of MDA by measuring TBARS (a product of the oxidative modification of lipids) in the plasma, hippocampus (HPC) and prefrontal cortex (PFC). TBARS levels were significantly increased in plasma, HPC and PFC of FSS mice compared to control mice (Plasma *p* < 0.01; HPC and PFC *p* < 0.05) (Figure 2A,C,E). Treatment with apocynin restored the normal levels of TBARS in stressed mice (Plasma *p* < 0.01; HPC and PFC *p* < 0.05) (Figure 2A,C,E). Remarkably, there was a significant positive correlation between NSF latency time and MDA levels only in the HPC (*r* = 0.62; *p* < 0.0001), but not in the PFC (*r* = 0.218; *p* = 0.36) or plasma (*r* = 0.24; *p* = 0.19) (Figure 2B,D,F). Therefore, we focused our following molecular analysis only on HPC.

### 3.3. Apocynin Treatment Prevented the Enhancement of Hippocampal p47phox Induced by FSS

**Figure 3:**
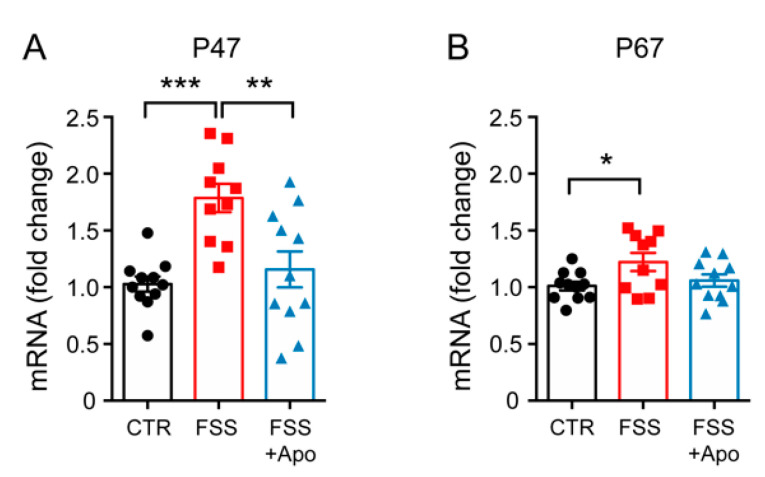
Apocynin prevents the FSS-induced increase of *p47phox* expression. (**A**,**B**) mRNA levels of *p47phox* (**A**) and *p67phox* (**B**) measured in the hippocampus (HPC). One-way ANOVA followed by Tukey’s post hoc analysis. Data are presented as mean ± SEM (*n* = 10–11 mice/group). * *p* < 0.05; ** *p* < 0.01; *** *p* < 0.001.

Because apocynin is an inhibitor of the activity and subunits expression of *Nadph* oxidase, we next evaluated the levels of *p47phox* and *p67phox*, two subunits of NADPH oxidase, in the HPC of the different groups of mice. mRNA levels of both *p47phox* and *p67phox* were increased in the FSS-exposed group compared to controls (*p47phox p* < 0.001; *p67phox p* < 0.05) (Figure 3A,B). Apocynin treatment significantly reduced only *p47phox* levels (*p* < 0.01) but not *p67phox* levels (*p* > 0.05) in the HPC of stressed mice.

### 3.4. Apocynin Treatment Prevented the Enhancement of Hippocampal Hdacs Induced by FSS

**Figure 4:**
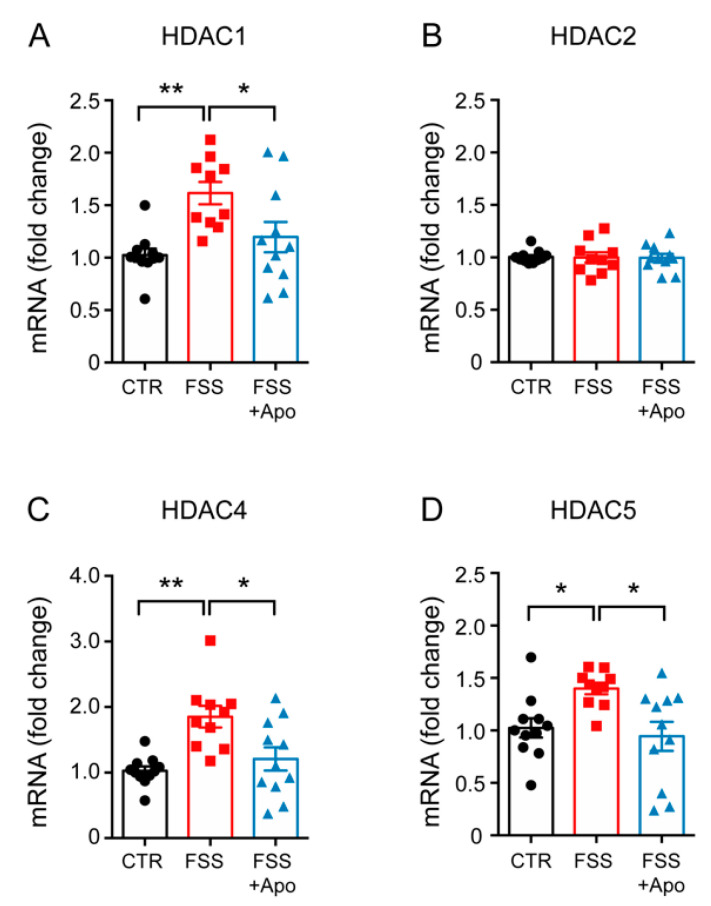
Apocynin prevents the FSS-induced increase of *Hdacs* expression. (**A**–**D**) mRNA levels of *Hdac1* (**A**), *Hdac2* (**B**), *Hdac4* (**C**), and *Hdac5* (**D**) measured in the hippocampus (HPC). One-way ANOVA followed by Tukey’s post hoc analysis. Data are presented as mean ± SEM (*n* = 10–11 mice/group). * *p* < 0.05; ** *p* < 0.01.

Next, we assessed if the expression of *Hdacs* were differentially modulated in the HPC of the different experimental groups. FSS enhanced the mRNA expression of *Hdac1* (*p* < 0.01), *Hdac4* (*p* < 0.01), *Hdac5* (*p* < 0.05) but not the expression of *Hdac2* in the HPC of vehicle-treated mice (Figure 4A–D). Apocynin administration restored the normal levels of *Hdac1* (*p* < 0.05), *Hdac4* (*p* < 0.05) and *Hdac5* (*p* < 0.05) in the HPC of stressed mice (Figure 4A–D).

### 3.5. Apocynin Treatment Prevented the Reduction of Hippocampal H3 Acetylation Induced by FSS

**Figure 5:**
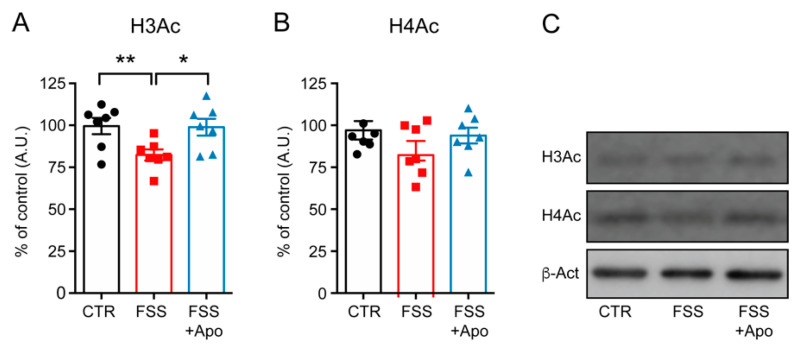
Apocynin prevents the FSS-induced reduction of H3 acetylation. (**A,B**) Levels of acetylated histone H3 (H3Ac) (**A**) and acetylated histone H4 (H4Ac) (**B**) measured in the hippocampus. Densitometric quantification were obtained as ratio of H3Ac/Actin and H4Ac/Actin. One-way ANOVA followed by Tukey’s post hoc analysis. Data are presented as mean ± SEM (*n* = 10–11 mice/group). (**C**) Representative Western blot images from H3Ac, H4Ac, and b-actin. * *p* < 0.05; ** *p* < 0.01.

To verify if the increase of *Hdacs* expression was paralleled by reduction of histone acetylation we measured the acetylation levels of histone H3 and H4 by Western blot analysis. FSS decreased the acetylation levels of histone H3 (*p* < 0.05) but not of H4 in the HPC (Figure 5A,B). Treatment with apocynin fully prevented the hippocampal reduction of H3Ac (*p* < 0.05) induced by FSS exposure (Figure 5A).

## 4. Discussion

In this work we found that administration of apocynin prevented the FSS-induced anxiety-like phenotype in mice. By studying the possible mechanisms responsible for this behavioral alteration, we observed that apocynin, a NADPH oxidase inhibitor, normalized increased lipid peroxidation levels caused by stress in the HPC, PFC and plasma. In addition, apocynin prevented the FSS-induced increases in the hippocampal levels of *p47phox* and *p67phox* as well as *Hdac1*, *Hdac4* and *Hdac5*. Finally, apocynin blocked the reduction of H3Ac levels promoted by subchronic stress exposure. Overall, these data suggest that NADPH-derived ROS may play a role in the susceptibility to develop anxious-like behavior after subchronic stress exposure, likely involving epigenetic mechanisms.

Consistent with our data, it was previously reported that treatment with apocynin prevented the depressive- and anxious-like phenotypes induced by chronic stress or corticosterone exposure [26,44,45].

Recent evidence suggests that brain oxidative stress is involved in the pathological changes induced by chronic stress. Indeed, it has been reported that chronic restraint stress enhanced MDA levels both in the HPC and PFC, while chronic mild stress increased MDA levels only in the ventral HPC, but not in the medial PFC [46]. On the other hand, chronic administration of CORT enhanced the production of ROS only in the PFC but not in the HPC [44]. Consistent with these studies, we found that four days of FSS were sufficient to increase MDA levels in the HPC, PFC and plasma. Altogether these results suggest that different types of stressors may differentially affect the level of oxidative stress in selected brain regions. Remarkably, we found a selective and significant correlation between hippocampal MDA levels and anxious-like phenotype. Although more studies are warranted, our data suggest that increased oxidative stress in the hippocampus may play a role in the behavioral alteration induced by FSS.

Several enzymes can produce ROS, such as xanthine oxidase, cytochrome P450 oxidases, lipoxygenases, NADPH oxidases, monoamine oxidases and the mitochondrial electron transport chain. However, most of these enzymes only produce ROS after they have been damaged by ROS. In contrast, NADPH oxidases produce ROS as their primary and sole function [47]. Remarkably, previous evidence showed that stress-induced oxidative damage in the brain occurs mainly through NADPH oxidase activity, a key enzyme involved in the generation of superoxides and related toxic metabolites [26,44,46]. Accordingly, we found that the pharmacological inhibition of NADPH oxidase, using the NADPH oxidase inhibitor apocynin, prevented the increase of MDA induced by four days of FSS in the plasma, HPC and PFC. However, apocynin treatment did not affect the stress-induced rise of corticosterone, suggesting that the physiological response to stress was not altered by apocynin administration.

To verify the possible mechanism of action of apocynin we analyzed the expression levels of *p47phox* and *p67phox*, two of the subunits of NADPH oxidase, in the hippocampus. We observed that *p47phox* and *p67phox* expression levels were raised in the stressed mice, while chronic apocynin treatment prevented such alterations. These variations were accompanied by similar changes in the hippocampal MDA levels, suggesting that apocynin can regulate NADPH activity by reducing p47phox and p67phox levels.

An open question is how apocynin may regulate the expression of *p47phox* and *p67phox*. Apocynin is recognized to be essentially a NADPH oxidase inhibitor and a ROS scavenger [31]. Moreover, the capability of apocynin to prevent Nuclear Factor-kappaB (NF-kB) activation has been also reported [48]. Oxidative stress has been recently recognized to play a key role in transcription of specific genes through the activation of redox-sensitive transcriptional factors such as Activator protein 1 (AO-1) Nuclear Factor *kappa* B (NF-κB), Specific Protein 1 (SP-1) and Hypoxia-Inducible Factor 1 (HIF-1) [49]. Remarkably, NF-κB and SP-1 can also regulate the expression of several *Nadph oxidase* subunits [50,51]. Therefore, it is possible to assume a positive feed-back mechanism in which apocynin, by decreasing the NADPH oxidase activity and ROS levels, reduces NF-kB and SP-1 transcriptional activities, thus reducing *p47phox* and *p67phox* expression. Future studies will be necessary to address this hypothesis.

The key role of *p47phox* in the stress-induced increase of oxidative stress and behavioral alterations haves been clearly demonstrated in heterozygous *p47phox*, which is resilient to stress-induced depressive and anxious-like phenotypes [25,26]. Intriguingly, molecular suppression of the specific *Nadph oxidase* subunits *gp91hox*, *Nox1* and *Nox2* was also reported to prevent stress-induced behavioral abnormalities [26,44,45].

Previously, we reported that apocynin prevented the prothrombotic phenotype induced by FSS exposure [38]. Indeed, oral apocynin administration reduced oxidative stress, number and maturation of megakaryocytes, amount of circulating leukocytes and platelets, and predisposition to arterial thrombosis induced by four days of FSS [38]. Our data presented here not only confirm the ability of apocynin to reduce the stress-induced ROS production in both the plasma and the brain, but also suggest that NADPH oxidase plays a key role in anxiety disorders induced by stressful events.

Altogether these results support the idea that the overactivation of NADPH oxidase induced by stress is a key player in both thrombosis and psychiatric disorders, and that apocynin could be proposed for the treatment of stress-related comorbid cardiovascular and mental illness.

Despite evidence that NADPH oxidase plays a key role in the onset of stress-related diseases, the mechanism(s) by which NADPH oxidase may induce an anxiety-like phenotype have not yet been clarified. One possible mechanism is that oxidative stress can promote alterations in gene expression through epigenetic changes [24]. In line with this hypothesis, our data showed that hippocampal expression levels of *Hdac1*, *Hdac4* and *Hdac5* were increased in response to FSS, and that treatment with apocynin can prevent these alterations. This also suggests that oxidative stress may regulate *Hdacs* expression. This hypothesis is corroborated by a previous in vitro study in which it was observed that acute treatment with H_2_O_2_ promoted the activity of HDACs class I and II, as well as reducing acetylating levels of H3K9Ac and H3K8Ac. Pretreatment with antioxidants, such as ascorbic acid, was able to prevent these alterations [52].

To the best of our knowledge, we showed here for the first time that apocynin reduced the over-expression of *Hdacs* induced by the FSS in the hippocampus. A possible mechanism by which apocynin may regulate the expression of *Hdacs* may be through the regulation of the Peroxisome Proliferator-Activated Receptor Gamma (PPARγ) transcriptional factor. PPARγ is a ligand-activated transcriptional factor that is broadly expressed and regulates several biological processes. The activity of PPARγ in a cell context relies on the presence of other coactivator and corepressor factors. Interestingly, it has been shown that PPARγ reduces the expression of the HDACs in carcinomas cells [53], and that the expression of PPARγ is reduced by oxidative stress [54]. Therefore, we can speculate that apocynin, by blocking the NADPH oxidase activity and by reducing ROS levels, may increase the expression of PPARγ, which in turn downregulates HDACs expression. Future studies will be required to explore this hypothesis.

Histone acetylation modification, controlled by HDACs, is an important mechanism regulating gene expression. The role of HDACs in stress responses has been extensively discussed in the literature and there is evidence suggesting that increased HDACs levels play a key role in the onset of psychiatric disorders [6,15,55,56,57]. Using our same FSS paradigm, Sailaja and colleagues found that hippocampal HDACs were upregulated in stressed-mice, and that pharmacological inhibition of HDACs, or specific molecular inhibition of HDAC4, reversed the stress-induced the anxious-like phenotype in mice, suggesting that down-regulation of HDACs, in particular HDAC4, has anxiolytic effects [39]. Another study highlighted the importance of HDAC5 in the action of antidepressants. It was shown that in mice over-expressing HDAC5, treatment with the antidepressant imipramine does not reverse the behavioral alterations induced by stress [14]. In line with the above-mentioned studies, we observed that the increase of *Hdac1*, *Hdac4* and *Hdac5* expression levels following FSS was paralleled by an anxious-like phenotype. In addition, the concomitant downregulation of *Hdacs* and increase of histone acetylation, associated with the reduction of the anxious-like phenotype induced by FSS, confirm the central role of HDACs in psychiatric disorders.

## 5. Conclusions

In conclusion, in this study we observed that apocynin prevents stress-induced molecular and behavioral changes, confirming the fundamental role of NADPH oxidase in the establishment of an anxiety-like phenotype and increased oxidative stress in the central nervous system induced by stress. Finally, we noted that increased oxidative stress levels are associated with increased *Hdacs* gene expression, suggesting that oxidative stress generated by stress may induce an anxious phenotype through epigenetic alterations. To support this hypothesis, further in vivo and in vitro studies will be necessary to confirm the direct causality between oxidative stress, epigenetic mechanisms, and behavioral changes.

## Figures and Tables

**Table 1:** Primer sequences.

Gene	Forward	Reverse
*p47phox*	ACCGGCTATTTCCCATCCAT	TGGATGCTCTGTGCGTTGC
*p67phox*	GCCAGCTTCGGAACATGGT	GACAGGAGCAGAAGCTCGTG
*Hdac1*	GAGTTCTGTCAGTTGTCCACGG	TTCAGACTTCTTTGCATGGTGC
*Hdac2*	GGGACAGGCTTGGTTGTTTC	GAGCATCAGCAATGGCAAGT
*Hdac4*	CAATCCCACAGTCTCCGTGT	CAGCACCCCACTAAGGTTCA
*Hdac5*	TGTCACCGCCAGATGTTTTG	TGAGCAGAGCCGAGACACAG
*Gapdh*	CGTGCCGCCTGGAGAAACC	TGGAAGAGTGGGAGTTGCTGTTG
*Rps18*	TGGAGCGAGTGATCACCATCA	CCTCACGCAGCTTGTTGTCTA

## Data Availability

The data presented in this study are available on request from the corresponding author.
